# Exercise-induced extracellular vesicles mediate apoptosis in human colon cancer cells in an exercise intensity-dependent manner

**DOI:** 10.1007/s00421-025-05787-1

**Published:** 2025-04-20

**Authors:** Berkay Ozerklig, Ibrahim Turkel, Merve Yilmaz, Refika Dilara Vaizoglu, Handan Sevim Akan, Z. Gunnur Dikmen, Ayesha Saleem, Sukran Nazan Kosar

**Affiliations:** 1 Department of Exercise and Sport Sciences, Faculty of Sport Sciences, Hacettepe University, Ankara, Türkiye; 2 Faculty of Kinesiology and Recreation Management, University of Manitoba, Winnipeg, Canada; 3 The Children’s Hospital Research Institute of Manitoba (CHRIM), Winnipeg, Canada; 4 Department of Medical Biochemistry, Faculty of Medicine, Hacettepe University, Ankara, Türkiye; 5 Department of Biology, Molecular Biology Section, Faculty of Science, Hacettepe University, Ankara, Türkiye; 6 Department of Biology, Faculty of Science, Hacettepe University, Ankara, Türkiye; 7 Division of Exercise Nutrition and Metabolism, Faculty of Sport Sciences, Hacettepe University, Ankara, Türkiye

**Keywords:** Exercise, Extracellular vesicles, Colon cancer, Apoptosis

## Abstract

Regular exercise reduces the incidence and improves the prognosis of many cancer types, but the underlying mechanisms remain elusive. Evidence suggests that exercise exerts its therapeutic effects through extracellular vesicles (EVs), which are essential for cellular communication. Here, we hypothesized that exercise-induced EVs from serum of healthy individuals would exert anti-tumorigenic effects on human colon cancer HT-29 cells, in an exercise intensity-dependent manner. Ten healthy young active males participated in a randomized crossover trial, completing two workload-matched acute exercise bouts, moderate-intensity continuous exercise (MICE) and high-intensity interval exercise (HIIE), on a cycle ergometer. A control session of rest (PRE) was included. EVs were isolated from serum samples collected during PRE and immediately after each exercise session. EVs were co-incubated with HT-29 colon cancer cells, and the effects on cell viability, migration, and apoptosis were measured. EV treatment reduced cell viability in all groups (PRE, MICE, and HIIE) by 35%, 43% and 47%, respectively, vs. PBS. HIIE-EVs showed a significantly greater reduction in cell viability vs. PRE; therefore, only these groups were used for further analysis. PRE EVs reduced migration by 27%, and HIIE-EVs by 39%. HIIE-EVs increased expression of pro-apoptotic markers: Bax/Bcl-2 ratio by 56% and Caspase 3 by 30% vs. PBS, with no change observed in the PRE group. Further, 16% of cells in PRE and 28% of cells in HIIE were TUNEL-positive, indicating DNA fragmentation. To our knowledge, this is the first human study that illustrates the therapeutic potential of exercise-induced EVs in cancer treatment.

**Graphical abstract:**

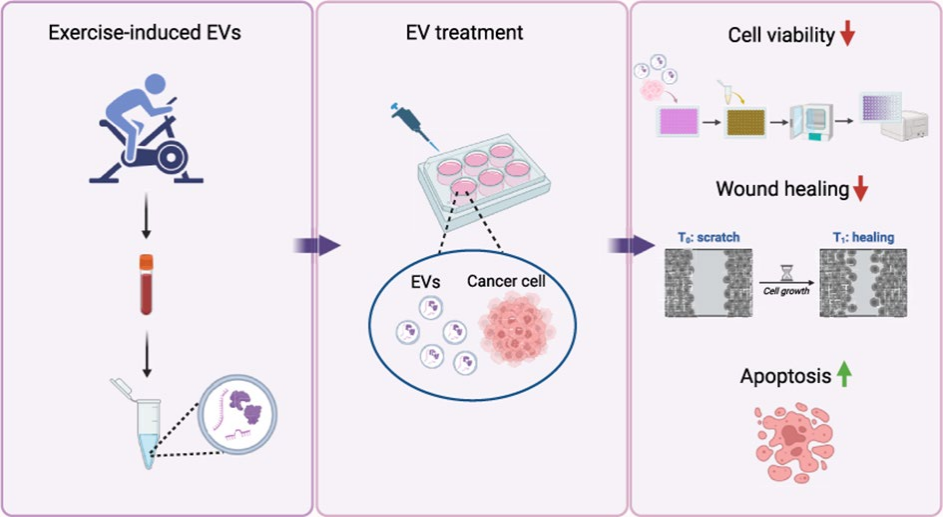

## Introduction

Colorectal cancer (CRC) is the third most prevalent cancer worldwide and the second leading cause of cancer-related mortality, with no definitive cure currently available (Morgan et al. 2023; Collaborators 2019; Sung et al. 2021). Evidence suggests that regular exercise can be protective against CRC in a dose-dependent manner (Patel et al. 2019; Matthews et al. 2020) and is associated with decreased cancer-related mortality and recurrence (McTiernan et al. 2019). Preclinical in vivo and in vitro studies demonstrate that systemic responses to exercise can inhibit cancer cell proliferation and metastasis (Hojman et al. 2011; Metcalfe et al. 2021). Exercise may exert these positive adaptations through the modulation of reactive oxygen species, inflammation, hormones, immune cell responses, and growth factors, which ultimately attenuate tumor formation through alterations in the tumor microenvironment and circulating factors (Ashcraft et al. 2016; Hojman et al. 2018). However, the exact biological mechanisms underlying these anti-carcinogenic effects of exercise have yet to be fully elucidated.

The beneficial systemic adaptations to exercise may, in part, result from factors released as a physiological response to exercise. Growing evidence suggests that these exercise-induced molecules, named “exerkines”—including cytokines, proteins, peptides, metabolites, and microRNAs (miRNAs)—are secreted from metabolically active tissues and exert pro-metabolic effects on multiple organs and biological processes in an autocrine, paracrine, and endocrine manner (Lee and Jun 2019; Magliulo et al. 2022; Chow et al. 2022). It follows that the transportation of these molecules to recipient tissues is essential for cellular communication and organ–organ crosstalk. Therefore, the mediators of such transport are pivotal. Exerkines can be released into circulation by themselves or can be packaged within extracellular vesicles (EVs) (Nederveen et al. 2020; Safdar et al. 2016).

EVs are released from all cells, play an essential role in cellular communication and carry biomolecular cargo that can affect recipient cell function. EVs are divided into different groups according to their sizes and classically labeled as exosomes (30–150 nm), microvesicles (100–1000 nm), and apoptotic bodies (500–5000 nm) (Doyle and Wang 2019). However, the Minimal Information for Studies of EVs (MISEV) 2023 guidelines recommend classifying EVs based on size (small EVs as < 200 nm and large EVs as > 200 nm), composition, and cell of origin (Welsh et al. 2024). Acute exercise increases circulating EV concentration and modifies EV cargo in both rats and humans (Annibalini et al. 2019; Whitham, et al. 2018). EVs isolated from healthy endothelial and stem cells have therapeutic effects on different disease models, both in vivo and in vitro, including ischemia–reperfusion injury in heart muscle (Yadid, et al. 2020), Alzheimer’s disease (Khan et al. 2023), chronic kidney disease (Wan et al. 2022), and CRC (Jahangiri et al. 2022). However, studies investigating the beneficial effects of exercise-induced EVs are sparse. A recent report showed that exercise-induced EVs attenuated tumor progression and metastasis in a sedentary syngeneic prostate tumor-bearing rat (Sadovska et al. 2021). Similarly, another recent study demonstrated that EVs isolated from murine skeletal muscle myotubes after chronic contractile activity, an in vitro model of exercise, perpetuated anti-tumorigenic effects in Lewis Lung Carcinoma cells (Obi, et al. 2024a), and had an altered EV proteomic cargo profile (Obi, et al. 2024b).

Here, we investigated the effects of exercise-derived EVs from healthy young active males on HT-29, a human colorectal adenocarcinoma cell line. We hypothesized that EVs isolated from participants immediately after acute exercise would inhibit cell growth by inducing apoptosis in HT-29 cells, in an exercise intensity-dependent manner. To evaluate this hypothesis, we used a randomized crossover experimental design with two different workload-matched exercise models: moderate-intensity continuous exercise (MICE) and high-intensity interval exercise (HIIE). Blood samples were collected at PRE, post-MICE, and post-HIIE time points, and serum extracted for EV isolation and analysis. EVs isolated from 250 µL serum samples were co-cultured with HT-29 cells (100 µg/µL, 48–72 h), and the effects on cell growth, viability, and apoptosis in recipient cells were measured.

## Methods

### Participants

Ten healthy recreationally active males (age: 25.4 ± 6.2 years) who engaged in physical activity 2–3 times per week were recruited. Participants were excluded if they had any known acute or chronic disease, smoking, or were taking any drug or supplements known to affect metabolism. Participants were given both oral and written information about the study procedures before they provided their written informed consent. The study was approved by the Hacettepe University Institutional Non-Interventional Ethical Committee (decision number: 2022/20–58) and conducted in accordance with the Helsinki Declaration.

### Experimental design

Using a randomized crossover design, participants were randomly assigned to either the MICE or HIIE group for the initial intervention. Participants visited the laboratory four times, each visit separated at least 48–72 h. On the first visit, body composition was measured, and a familiarization session was performed to accustom subjects to the equipment, maximal oxygen consumption (VO_2_max) test, and exercise interventions (MICE and HIIE). On the second visit, participants performed a progressive incremental exercise test on a bicycle ergometer to determine their respective VO_2_max. On the third and fourth visits, each participant performed a single session of either MICE or HIIE. Venous blood samples were collected at three time points: (1) immediately before the first exercise session (PRE), (2) immediately after the MICE session, and (3) immediately after the HIIE session. Blood samples were used for serum and subsequently EV isolation as described below. To minimize the influence of circadian rhythms, all exercise intervention protocols were performed at the same time of day (13:00–17:00 h). Participants were instructed to maintain their usual exercise and dietary habits, but were asked to abstain from coffee and alcohol, and avoid strenuous exercise for 24 h prior to the acute MICE and HIIE sessions. Upon arrival at the laboratory, participants were asked to consume a 600 kcal standardized meal and perform a single MICE or HIIE session 2 h after the meal.

### Body composition

Body composition of each participant was measured using dual-energy X-ray absorptiometry (DXA, Lunar Prodigy Pro Narrow Fan Beam (4.5°), GE Health Care, Madison Wisconsin, USA) after an overnight fast and following standardized procedures (Nana et al. 2013). Body fat mass (kg), percent body fat (%), lean body mass (kg), and fat-free mass (kg) were analyzed by GE enCORE™ v.17.0 software (GE Healthcare, Madison, WI).

### Determination of maximal oxygen uptake

All participants underwent an incremental exercise protocol on a bicycle ergometer (COSMED E200, Italy) using breath-by-breath technology (Quark CPET, COSMED Cardio-Pulmonary Exercise Testing, Italy) to determine individual VO_2_max. Briefly, the exercise protocol started with a load of 60 W, which was increased by 30 W every 2 min. After the completion of three phases at 60, 90, and 120 W, the load was further increased by 30 W per min. Throughout the protocol, participants were instructed to maintain a stable, self-selected cadence of approximately 70 rpm for the duration of the test (Jeukendrup et al. 1996). The test was terminated when at least two of the following criteria were met: (1) oxygen levels plateaued even with increasing workload, (2) the respiratory exchange ratio exceeded 1.1, (3) the heart rate was within 10 beats per min (bpm) of the predicted maximum (220-age), and (4) unable to maintain 70 rpm for 10 s despite verbal encouragement (Howley et al. 1995).

### High-intensity interval exercise (HIIE) protocol

After a 3 min warm-up at 60 W, participants performed 10 × 1 min intervals at 90% of VO_2_max, separated by 75 s of active recovery at 60 W and a final 3 min cool down period at 60 W (Little et al. 2010).

### Moderate-intensity continuous exercise (MICE) protocol

Similar to the HIIE group, participants performed a single session of MICE matched to the HIIE workload under supervision on a bicycle ergometer. Based on power output during HIIE session, the MICE group completed the ~ 21 min of exercise at 55% of VO_2_max consisting of a 3 min warm-up and 3 min cool down at 60 W.

Although the literature lacks a clear approach on standardizing parameters for the two different exercise protocols, previous studies suggest equalizing total workload (total exercise volume) and energy expended (isocaloric) (Sadovska et al. 2021). Therefore, in this study, total workload equalization was preferred. Heart rate (HR) was continuously monitored throughout the exercise sessions using a heart rate monitor (Garmin International Inc., Olathe, KS, USA).

### Blood sampling and EV isolation

Venous blood samples were collected from antecubital vein before and after the completion of each acute exercise session as described above in serum separating tubes (BD Vacutainer). Approximately, 5 mL of blood was collected, allowed to clot for 40 min, and then centrifuged at 2000*xg* for 10 min at 4 °C, with resulting supernatant collected as serum. Serum samples were aliquoted and stored at – 80 °C until EV isolation. EVs were isolated from serum samples using ExoQuick Serum Exosome Precipitation Solution (System Biosciences, Palo Alto, CA, USA) according to manufacturer’s instructions and as before (Taylor et al. 2011). EV preparations were aliquoted and conserved at − 80 °C for later use.

### NTA measurement with Nanosight NS300

EVs were characterized biophysically using the NanoSight NS300 Instrument (405 nm laser diode) according to the manufacturer’s protocols (Malvern Instruments Inc.) and in accordance with MISEV guidelines (Welsh et al. 2024; Bachurski et al. 2019). The instrument was calibrated prior to each experimental run for nanoparticle size and quantity using standardized nanoparticle dilutions provided by the manufacturer. Suitable dilution of isolated EVs was defined before each measurement. EVs diluted by 1:1000 in sterile PBS and analyzed by NanoSight NS300. Each experimental run was performed in quintuplicate (each capture 60 s) and PBS was used to assess background. For each sample, the nanoparticle size distribution curve, refractive index, and the relative nanoparticle concentration of a particular size was recorded, with the cumulative percentage of nanoparticles.

### Transmission electron microscopy (TEM)

10 μL of 1:1000 diluted EVs in PBS was fixed with an equal volume of 4% paraformaldehyde and carefully applied onto a copper grid for 20 min at room temperature. Grids were washed with 100 μL PBS and fixed with 1% glutaraldehyde for 5 min. To remove the excess fixation buffer, the grids were washed 8 times with 100 μL distilled water, after which they were negatively stained with 1% uranyl acetate for 2 min and left to air dry at room temperature. TEM grids were visualized using a JEM-2100 F field emission electron microscope operated at 80 kV using the fee-for-service at METU Central Lab (R&D Department TEM Laboratory, Middle East Technical University).

### EV protein extraction and quantitation

EV preparations were lysed using RIPA solution with protease inhibitor tablet (Roche) and EV concentration was determined using Pierce™ BCA protein assay kit (ThermoFisher Scientific) following manufacturer’s instructions and as before (Saleem et al. 2015). Briefly, the working reagent was prepared by mixing 24 parts of Reagent A, and 1 part of Reagent B. Standards were prepared by serial dilution of 2 mg/mL bovine serum albumin (BSA) ampule into clean vials using ddH_2_O. 25 μL of each standard or sample was added to a 96-well microplate in duplicates, followed by the addition of 200 μL of working reagent to each well. Samples were incubated at 37 °C for 30 min and absorbance was measured at 562 nm using a microplate spectrophotometer (SpectraMax i3x, Molecular Devices, USA).

### Cell culture and EV treatment

HT-29 human colon cancer cells were a gift from Professor Z. Gunnur Dikmen (Hacettepe University) and originally purchased from American Type Culture Collection (ATCC, HTB-38). Cells were cultured in growth media made with fresh Dulbecco’s Modification of Eagle’s Medium (DMEM; Sigma-Aldrich) supplemented with 10% fetal bovine serum (FBS; Gibco/ThermoFisher Scientific) and 1% penicillin/streptomycin (P/S). Cells were grown at 37 °C in 5% CO_2_ incubator for 24 h. Cells were seeded in 96-well microplates for the MTT assay, and 24-well or 6-well plates for all other experimental outcomes. Cells were incubated at 37 °C in a humidified atmosphere containing 5% CO_2_ for 24 h before proceeding with EV treatment, as described below for each outcome variable.

### Assessment of cell viability

The effect of EVs on cell proliferation and viability were assessed by the colorimetric 3-(4,5-dimethylthiazol-2-yl)−2,5-diphenyltetrazolium bromide (MTT) assay as described previously (Riss et al. 2016). MTT was purchased from Millipore Sigma (Milan, Italy). For this assay, 9,000 cells/well were seeded in a 96-well plate and co-cultured with 100 µg/µL EVs for 48 h. MTT solution (1 mg/mL) was added to the cell medium, and cells incubated for 2 h at 37 °C in the dark. MTT is metabolized to formazan salt in viable cells (Kumar et al. 2018). Thus, cell viability was determined by analyzing the absorbance of the formazan read at 490 nm wavelength using a microplate spectrophotometer (SpectraMax i3x, Molecular Devices, USA).

### Cell scratch assay

Cell migration was assessed using a scratch wound healing assay (Liang et al. 2007). HT-29 cells (2 × 10^5^ cells/well) were seeded in 24-well plates to grow in a monolayer for 24 h. After treating the cells with EVs (100 µg/mL), a sterile 20–200 μL pipette tip was held vertically to scratch a cross in each well. The scratch closure was monitored and imaged in 0, 24 and 48 h after scratch. The gap distance was quantitatively evaluated using ImageJ (National Institutes of Health). Cell migration was measured without inhibiting proliferation.

### HT-29 cell protein extraction and western blotting

To evaluate the anti-tumorigenic effects of exercise EVs, we focused on the expression of intrinsic apoptosis pathway-related proteins such as Bax, Bcl-2 and Caspase 3 (Qian et al. 2022), as well as an early marker of DNA double-strand breaks, γH2 AX^Ser139^ (Paull et al. 2000). In addition, markers of EV sub-types were analyzed in line with international standardized guidelines for EV research (Welsh et al. 2024). Briefly, HT-29 cells were trypsinized and seeded at 6-well plates (6 × 10^5^ cells/well). Upon reaching to 80% confluence, cells were gently washed with PBS and treated with 100 µg/mL EVs in fresh media for 72 h. A longer time point was used to precisely evaluate the changes in protein expression and apoptotic induction. After 72 h, cells were lysed using ice-cold RIPA buffer with protease/phosphatase inhibitor (Roche) for 30 min and centrifuged for 15,000xg for 30 min. Supernatants were transferred in a new tube and protein concentration was determined using Pierce™ BCA protein assay kit (ThermoFisher Scientific) following the manufacturer’s instructions.

10–30 μg of total protein from HT-29 cell extracts was resolved on a 10–12% SDS-PAGE gel and subsequently transferred to PVDF membranes. Membranes were then blocked for 1 h with 5% skim milk in 1x Tris-buffered saline-Tween 20 solution (TBST) at room temperature, followed by incubation with primary antibodies in 3% skim milk overnight at 4 °C. The following primary antibodies were used: rabbit monoclonal anti-CD63 (ab275018, Abcam, 1:1000) and TSG101 (ab275018, Abcam, 1:1000) for EV characterization; rabbit polyclonal anti-Bax (A20227, ABclonal, 1:1000), anti-Bcl-2 (A0208, ABclonal, 1:1000), anti-Caspase-3 (A0214, ABclonal, 1:1000), and anti-p-H2 AX^Ser139^ (#9718, Cell Signaling Technologies, 1:1000). Subsequently, membranes were washed three times for 5 min with TBST, followed by incubation with anti-rabbit (AS014, ABclonal) IgG HRP conjugated (1:1000–10000) in 3% skim milk for 1 h at room temperature. After the membranes were washed, a chemiluminescence system (ChemiDoc™, Bio-Rad Laboratories) was used to detect labeled proteins. Quantification of the bands from the immunoblots was performed using computerized densitometry software ImageJ (National Institutes of Health, Bethesda, MD).

### TUNEL assay

TUNEL detection kit (Elabscience, E-CK-A322) was used for assessing DNA fragmentation, the hallmark measurement of apoptosis, after EV treatment. Briefly, 50,000 cells/well were seeded in 24-well plates and allowed to adhere for 6 h, then co-incubated with 100 µg/mL EVs in fresh media for 72 h. Treated HT-29 cells were fixed with 4% paraformaldehyde for 15 min and incubated with 1% Triton X-100 for 10 min at 37 °C. After TUNEL staining, HT-29 cells were immersed in DAPI solution to stain cell nuclei and washed three times with PBS. We used an Olympus (Olympus, Japan) IX70 inverted fluorescence microscope to acquire the images. The images were analyzed by ImageJ (National Institutes of Health, Bethesda, MD).

### Statistical analyses

All data were analyzed using paired Student’s t test or one-way ANOVA. Multiple comparisons in the one-way ANOVA were corrected using Tukey’s post hoc test. Individual data points are plotted, with mean ± standard error of mean (SEM) shown as applicable. All graphs were created using GraphPad-Prism software (version 10.1.2, GraphPad, San Diego, CA, USA). Significance was set at *p* < 0.05. Exact *p* values are given for significant or close to statistically significant results. Experiments were conducted with a sample size of *n* = 4–10.

## Results

### Participant demographics

**Fig. 1:**
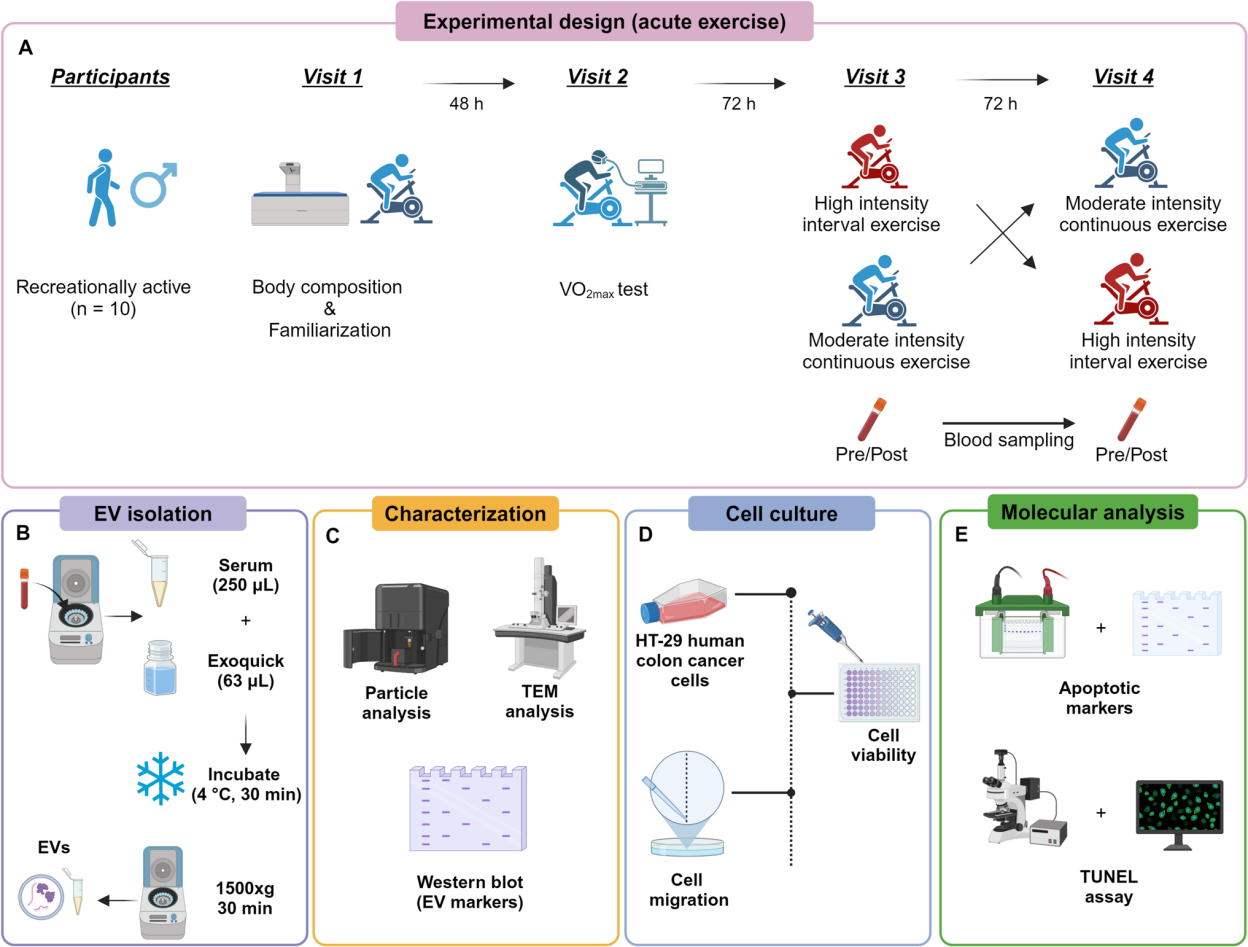
Experimental design of the study. **A** Recreationally active participants were recruited and provided written informed consent. All the participants attended the laboratory four times. In the first visit, participants underwent a DXA scan to determine body composition and were familiarized with the equipment. In the second visit, a VO_2_max test was performed to calculate exercise intensities for HIIE or MICE intervention. Participants were then randomly allocated to HIIE or MICE groups, and completed each exercise intervention in the third and fourth visits as part of a randomized crossover design. Blood samples were taken immediately before (PRE) and after exercise tests (post). **B** EV isolation from serum samples of participants, **C** EV characterization with NTA, TEM, and western blotting was done in line with standardized guidelines for EV research. **D** Cell viability and cell migration experiments with HT-29 cells treated with EVs, as well as **E** Western blot analysis for pro-apoptotic proteins and TUNEL assay for measuring DNA fragmentation in HT-29 cells treated with EVs were performed. Figure created using BioRender.com

The overall study design and the participants’ characteristics are shown in Fig. 1 and Table 1, respectively. Participants were 25.4 ± 6.2 years old, with a BMI of 23.5 ± 1.2 kg/m^2^, body fat of 19.6 ± 4.9%, and fat-free mass of 60.3 ± 7.3 kg (Table 1). The participants had an average VO_2_max of 45 ± 3.7 mL/kg/min (Table 1), which is considered an above average/good score for males of this age (Vaisanen et al. 2024). Combined with the 19.6 ± 4.9% body fat, which is within the healthy range for this age group (Mattila et al. 2007), this indicates that the participants were physically fit with good cardiorespiratory fitness levels.

**Table 1:** Participants’ characteristics (n = 10)

Characteristics	Mean ± SD
Age (years)	25.4 ± 6.2
Body mass (kg)	73.4 ± 6.1
Height (cm)	176.9 ± 6.9
Body mass index (kg/m^2^)	23.5 ± 1.2
Lean mass (kg)	57.2 ± 6.9
Fat mass (kg)	14.2 ± 3.7
Fat-free mass (kg)	60.3 ± 7.3
Percent body fat (%)	19.6 ± 4.9
VO_2_max (mL/kg/min)	45 ± 3.7

Abbreviations: VO_2_max, maximal oxygen consumption

### Biophysical characterization of EV preparations and effect of exercise on small EV protein yield

**Fig. 2:**
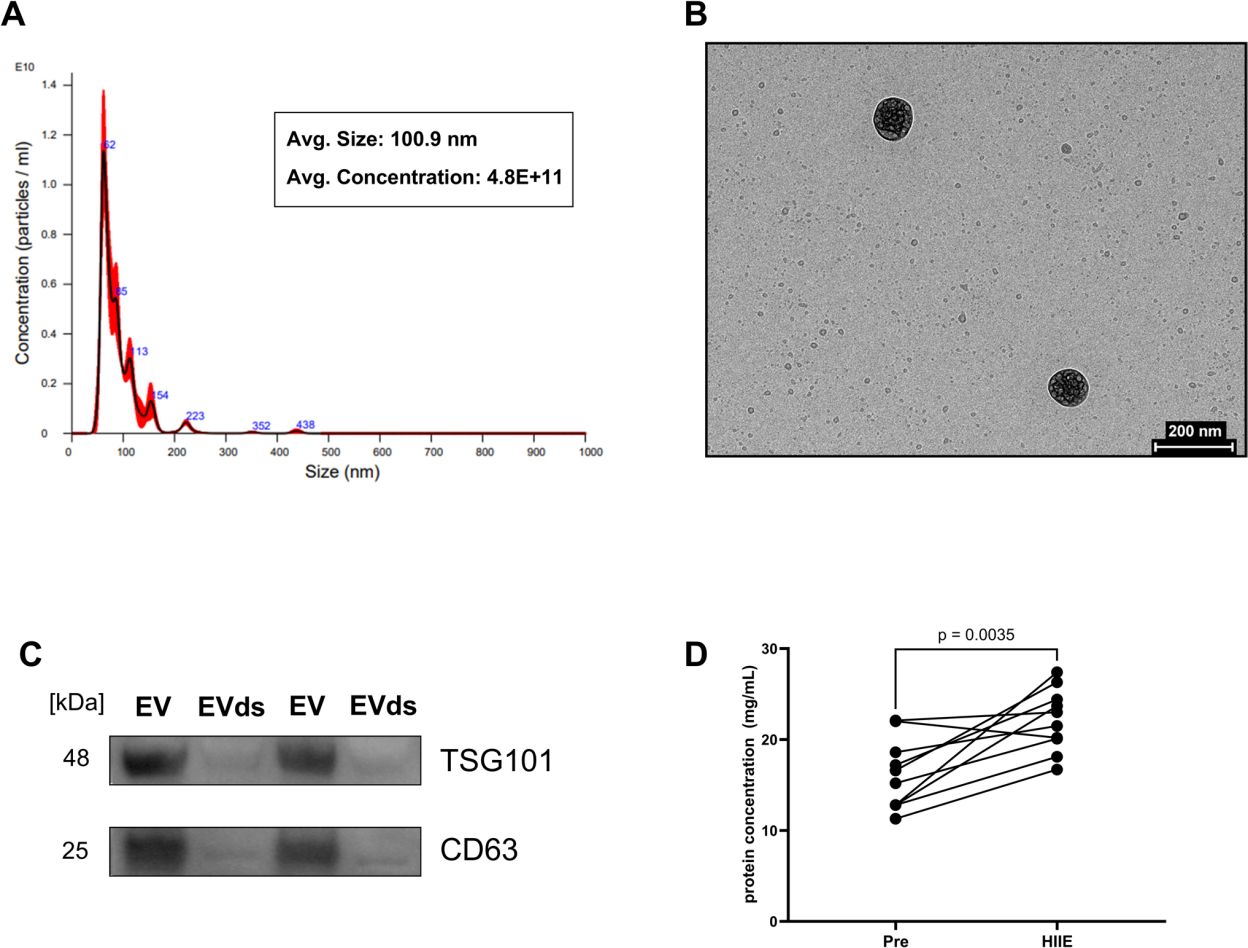
Characterization of EVs. EVs were isolated from 250 µL serum and characterized using nanoparticle tracking analysis (NTA), transmission electron microscopy (TEM), and Western blotting. **A** Representative NTA analysis graph showing size distribution and concentration of EVs isolated from human serum samples with average EV size and concentration data (*n* = 3). We observed an enrichment of small EVs (< 200 nm) in our EV preparations. **B** Representative TEM image, and **C** expression of small EV markers TSG101 and CD63 in isolated EVs and EV-depleted (EVds) serum samples further confirmed the enrichment of small EVs in isolated EV fractions in the study. **D** Protein concentrations of EVs isolated from PRE and HIIE serum samples showed an increase in protein yield in HIIE group vs. PRE (*p* = 0.0035, *n* = 10). Data in 2D were analyzed using a paired Student’s t test. Significance was set at *p* < 0.05

Isolated EVs had an average size of 100.9 nm and an average concentration of 4.8E + 11 particles/mL (*n* = 3, Fig. 2A). TEM images demonstrated the isolation of intact double-membraned vesicles, roughly ~ 100 nm in size (Fig. 2B). Small EV markers TSG101 and CD63 were enriched in EV preparations (Fig. 2C). EV-depleted serum (EVds) was used as a negative control and had significantly depleted expression of small EV protein markers. Altogether, EV characterization experiments illustrate that small EVs were successfully isolated. Finally, we noted that EV protein yield was increased in HIIE condition vs. PRE (*p* = 0.0035, *n* = 9, Fig. 2D).

### Exercise-induced EVs decrease cell viability and proliferation/migration in HT-29 cells

**Fig. 3:**
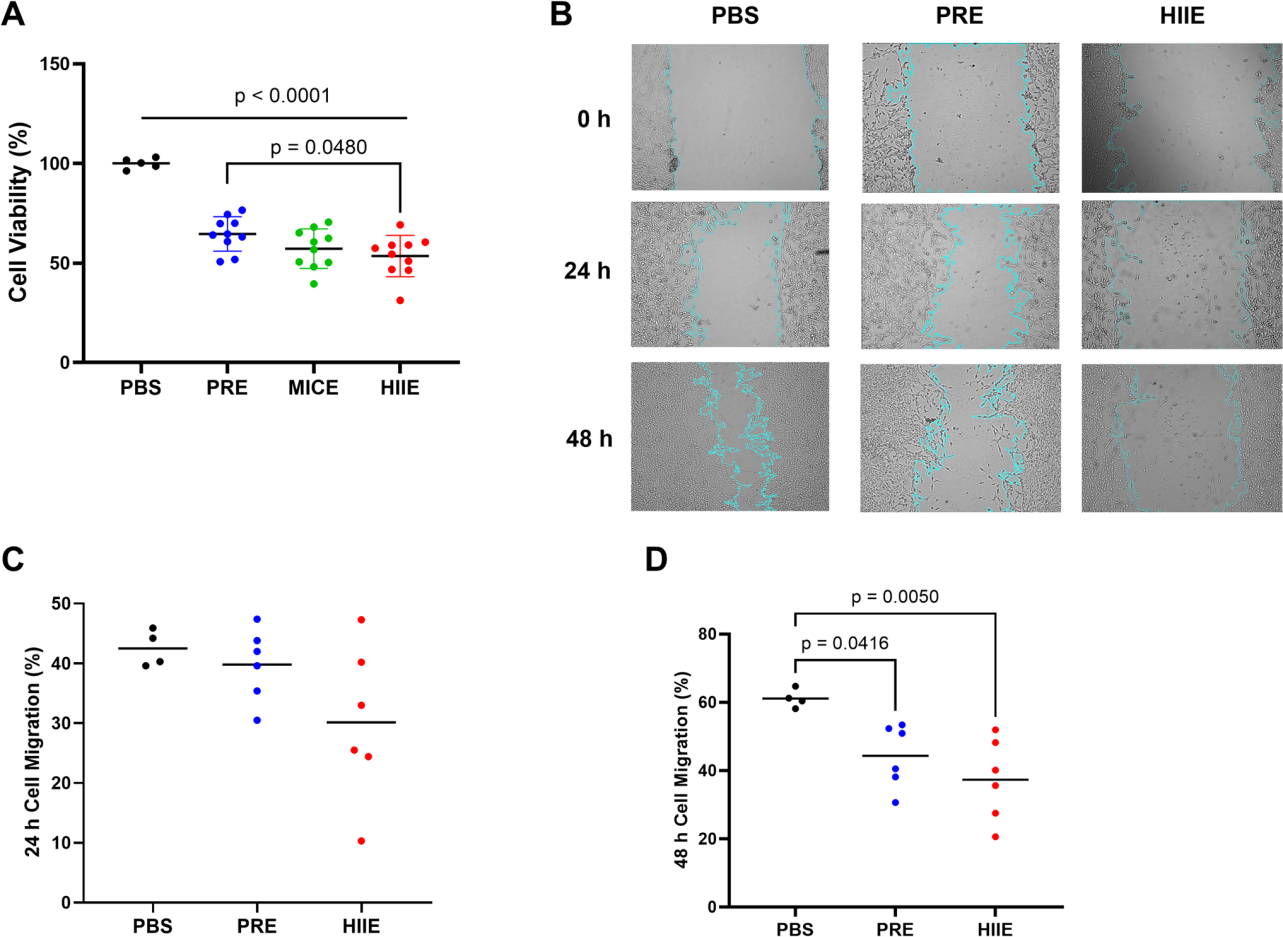
Cell viability and scratch assay. HT-29 colon cancer cells were treated with EVs (100 µg/mL) isolated from the different groups to elucidate if EVs could modulate cancer cell viability and migration. **A** Cell viability decreased in all EV-treated groups compared to PBS as determined using the MTT assay, in PRE by 35%, in MICE by 43%, and in HIIE by 47% (*p* < 0.001, *n* = 5–10 per group). Cells treated with EVs isolated from the HIIE group showed a further 20% decrease in cell viability compared to cells treated with EVs from the PRE condition (*p* = 0.0480, *n* = 5–10). **B** HT-29 cells were seeded in 24-well plates and kept in a 37 °C CO_2_ incubator for 24 h before EV treatment. Representative images from a cell scratch assay at 0, 24, and 48 h post-PBS or EV treatment, with quantification of data in panels **C** and **D**. **C** After 24 h of EV treatment, there was no difference between the groups. **D** After 48 h, cells treated with EVs from the PRE and HIIE groups showed decreased cell migration compared to PBS by 29%, (*p* = 0.0416, *n* = 4–6), and 39%, respectively (*p* = 0.0050, *n* = 4–6). Data were analyzed using one-way ANOVA with Tukey’s post hoc test. Significance was set at p < 0.05. The p values for non-significant data are not shown

The effect of exercise-induced EVs on HT-29 cell viability was measured with an MTT assay. The results showed that cell viability decreased in all EV-treated groups compared to PBS: in PRE group by 35%, in MICE group by 43%, and in HIIE group by 47% (*p* < 0.0001, *n* = 5–10 per group, Fig. 3A). In addition, cells treated with EVs isolated from the HIIE group showed a 20% decrease in cell viability compared to cells treated with EVs from the PRE group (*p* = 0.0480, *n* = 5–10 per group, Fig. 3A). As the HIIE group exhibited a significant reduction in cell viability compared to the PRE group, only this group was utilized as the exercise group for the remaining analyses. Next, we measured cell migration with a cell scratch assay, with the caveat that since cell growth was not controlled, the effects could be due to a combination of migration and cell proliferation. No significant change was observed after 24 h of incubating the HT-29 cells with EVs (Fig. 3C), however, at the 48-h time-point cells treated with EVs from the PRE and HIIE groups showed decreased cell migration compared to PBS by 29%, (*p* = 0.0416, *n* = 4–6, Fig. 3D) and 39%, respectively (*p* = 0.0050, *n* = 4–6, Fig. 3D).

### Exercise-induced EVs induce apoptosis in HT-29 cells

**Fig. 4:**
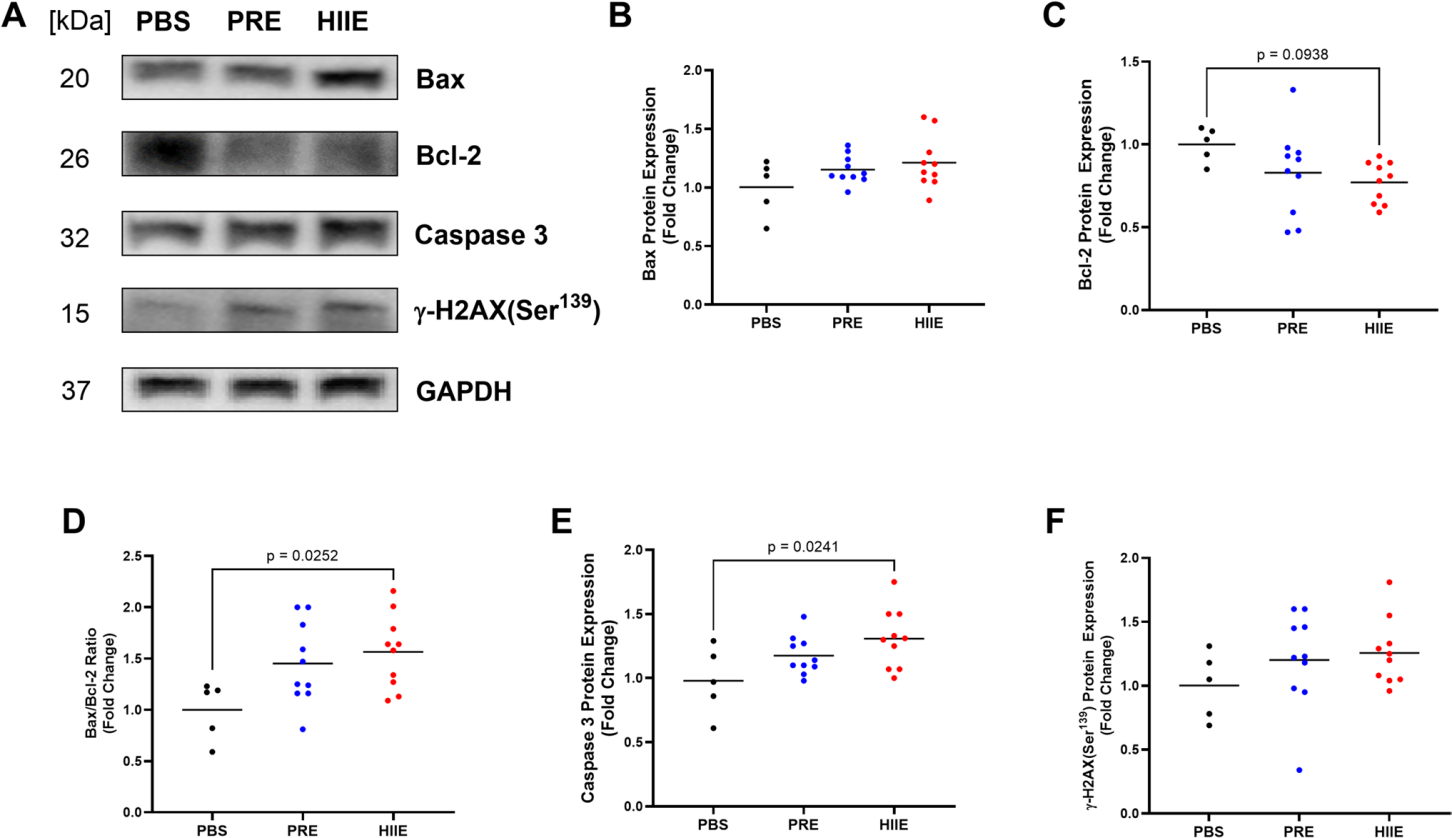
The expression levels of pro-apoptotic proteins in HT-29 cells after 72 h of EV treatment. Cells were treated with PRE and HIIE-EVs for 72 h, lysed, proteins extracted, and then resolved on a 10–12% SDS-PAGE for protein separation and Western blotting analysis. GAPDH was used as a loading control. **A** Representative immunoblots illustrating the expression of pro-apoptotic proteins in HT-29 cells treated with EVs from PRE, HIIE conditions, or PBS. There was no difference in protein levels of **B** Bax, **C** Bcl-2, and **F** γ-H2 AX between the groups. **D** Bax to Bcl-2 expression ratio increased by 57% (*p* = 0.0252, *n* = 6–10), and **E** Caspase 3 expression increased by 33% (*p* = 0.0241, n = 6–10) in cells treated with EVs from HIIE compared to PBS. Data were analyzed using one-way ANOVA corrected with Tukey’s post hoc test. Significance was set at *p* < 0.05. The p values for non-significant data are not shown

To gain a deeper insight into the observed decrements in cell viability and migration with EV treatment, we measured expression of proteins involved in the intrinsic apoptotic pathway. Representative immunoblots for Bax, Bcl-2, Caspase 3, yH2 AX (Ser139), and GAPDH as loading control are shown in Fig. 4A, as well as quantification of results in Fig. 4B-F, while there were no statistically significant alterations in Bax (Fig. 4B) and Bcl-2 (Fig. 4C) expression with EV treatment for PRE or HIIE. However, EV treatment from HIIE group increased Bax/Bcl-2 ratio by 57% (*p* = 0.0252, *n* = 6–10, Fig. 4D) and Caspase 3 expression by 33% (*p* = 0.0241, *n* = 6–10, Fig. 4E). yH2 AX-Ser139 expression remained unchanged (Fig. 4F).

**Fig. 5:**
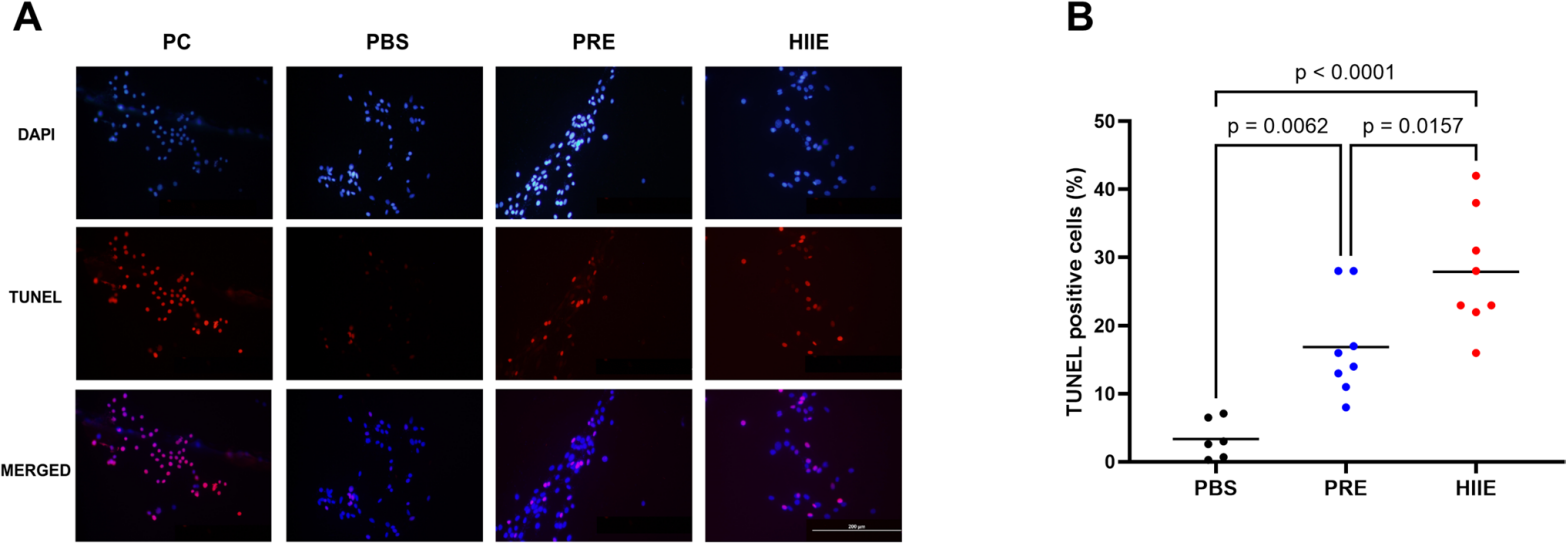
Evaluation of apoptosis in HT-29 cells treated with EVs using the TUNEL assay. HT-29 colon cancer cells were treated for 72 h with EVs (100 µg/mL) isolated from PRE and HIIE conditions to elucidate if EVs could modulate apoptosis in cancer cells. DAPI was used to stain the cell nuclei. Scale bar = 200 µm. Apoptotic cells (TUNEL-positive, cells indicating DNA fragmentation, a hallmark feature of apoptosis) in four random fields were counted and used for apoptotic analysis. **A** Representative images of HT-29 cells incubated with EVs from PBS or PRE and HIIE groups for 72 h. **B** EVs isolated from human serum samples induced apoptosis by 16% in PRE (*p* = 0.0062, *n* = 6–8) and by 28% in HIIE (*p* < 0.0001, *n* = 6–8) compared to PBS. Data were analyzed using one-way ANOVA corrected with Tukey’s post hoc test. Significance was set at *p* < 0.05. The p values for non-significant data are not shown

Finally, to ascertain if the HT-29 cells are undergoing apoptosis, we measured DNA fragmentation, a hallmark feature of apoptosis, using a TUNEL assay. Representative images (Fig. 5A) and quantification of data are shown in Fig. 5B. EVs isolated from human serum samples increased TUNEL-positive nuclei by 1.6-fold in PRE group (*p* = 0.0062, *n* = 6–8, Fig. 5B) and by 2.8-fold in HIIE (*p* < 0.0001, *n* = 6–8, Fig. 5B) compared to PBS. Altogether, exercise-induced EVs have a pro-apoptotic effect on HT-29 cells.

## Discussion

Here, we sought to investigate the effects of exercise-induced EVs on cell viability, migration/proliferation, and apoptosis in HT-29 human CRC cells. To our knowledge, this is the first investigation into the biological effects of human exercise-derived EVs on cancer cells. We found that exercise EVs, particularly those isolated from serum of health young males after high-intensity interval exercise, reduced cell viability, mitigated migration/proliferation and induced intrinsic apoptosis culminating in increased DNA fragmentation, a hallmark measurement of apoptosis, in HT-29 cells.

We isolated EVs that were intact, enriched with small EVs as corroborated by EV size, TEM image, and protein marker expression data, in compliance with MISEV recommendations (Welsh et al. 2024). The measured EV concentration in our study was in line with estimated plasma EV concentrations as reported previously (Johnsen et al. 2019). Interestingly, we saw a 43% increase in serum EV protein yield in HIIE compared to PRE, in line with previous reports showing acute exercise increases EV concentration and/or EV protein yield (Nederveen et al. 2020). For EV treatment, we selected an EV dose that seemed effective in a previous work (Patel et al. 2019) and used the same dosage for each condition (PRE, MICE, HIIE). We recognize that given the increase in circulatory EVs with exercise in humans, the physiological dosage may be higher in vivo. EV treatment significantly reduced cell viability and migration/proliferation in all EV-treated groups, including PRE EVs. A reduction in cell viability and migration/proliferation in the PRE group was unexpected. We posit that this is likely due to the above average cardiorespiratory fitness levels (average of ~ 45 mL/kg/min VO_2_max, low body fat%) of our participants. We observed an additive decrease in cell viability, migration, and/or proliferation in the HIIE group vs. PRE, but not with MICE group, and subsequently, used PRE and HIIE groups for all further analyses. This additive effect can be attributed to the difference in exercise intensities of the two protocols. Indeed, in the present study, the maximum heart rates achieved during the MICE and HIIE protocols were 166.4 ± 10.1 and 178.5 ± 10.8 beats/min, respectively (data not shown). Given that heart rate is a direct indicator of exercise intensity, it is anticipated that HIIE would lead to increased physiological stress and thus higher levels of circulatory exerkines. The exercise intensity-dependent effect is corroborated by previous reports where moderate-to-vigorous intensity acute exercise-conditioned serum exerts suppressive effects on different cancer cell lines including colorectal cancer cell lines (Bettariga et al. 2024). It is postulated that this anti-tumorigenic effect is caused by an increase in exerkines, including SPARC, OSM, and IL-6 (Bettariga et al. 2024). Cell migration is linked with cancer metastasis (Stuelten et al. 2018), thus inhibiting or attenuating cell migration is important for cancer treatment and this experiment is used frequently in cancer therapeutic research. Interestingly, we observed a significant reduction in cell migration in both PRE and HIIE groups at the 48 h time point, in parallel with our cell viability results. A recent study showed that exercise-conditioned serum derived from cancer patients inhibited the cell migration of pancreatic cancer cells (Tai, et al. 2024). Similarly, exercise-induced EVs isolated from rats attenuated the metastasis in sedentary tumor-bearing counterparts (Sadovska et al. 2021). Since we did not inhibit cell proliferation while completing the scratch assay experiments, our results could be due to either migration or proliferation of cells. Future work where cell growth is inhibited by mitomycin C (Obi, et al. 2024a) can identify if the anti-migration effects are due to a reduction in cell movement or cell growth or both.

Next, we examined upstream protein mediators and end stage marker of apoptosis in CRC cells with EV treatment. Apoptosis is an essential programmed cell death pathway and can be induced either intrinsically or extrinsically (Elmore 2007). We observed a trend towards an increase in pro-apoptotic protein Bax content, concomitantly with a trend towards a decrease in anti-apoptotic Bcl-2 expression (*p* = 0.09), with a significant increase in the overall Bax/Bcl-2 ratio, a hallmark indicator of apoptosis, in the HIIE treatment. Similarly, Caspase 3 expression increased significantly in HIIE treatment, which is a well-known initiator for DNA fragmentation in apoptosis. We measured γ-H2 AX^(ser139)^ as a marker for double-strand DNA breaks, but did not see a significant increase with HIIE EV treatment. It is likely that a temporal increase in phosphorylation of γ-H2 AX^(ser139)^, an early marker of DNA damage, was not captured in our experimental end time of 72 h. Shorter time courses may reveal temporal changes in the induction of the upstream mediators of the intrinsic apoptotic pathway. Interestingly, similar to our cell viability/migration and western blot findings, we observed a significant increase in TUNEL-positive nuclei, a hallmark marker for late-stage apoptosis, in both PRE and HIIE EV-treated cells. Like with cell viability results, HIIE group had additive increase in the number of TUNEL-positive nuclei compared to PBS and PRE groups. Overall, it is interesting to see that exercise-induced EVs induce apoptosis in HT-29 cells.

Our study has a few limitations. First, we did not treat healthy non-cancer cells with the different EVs, so we cannot deduce if the HIIE EV treatment will reduce cell viability, migration/growth, and induce apoptosis in all cells or if the effect is specific to CRC cells. Two recent reports demonstrated that EVs released from C2 C12 murine muscle myotubes following chronic contractile activity, an in vitro model exercise, induced a beneficial increase in mitochondrial biogenesis and elevated oxygen consumption rates (Obi, et al. 2024c) when incubated with C2 C12 myoblasts, but induced cancer cell senescence, cell death and reduced viability when cultured with LLC cells (Obi, et al. 2024a). This intriguing observation brings up a question as to whether exercise EVs might have different effects on different cell types, and the underlying mechanisms dictating recipient cell response to EV treatment. However, these questions require further investigation to elucidate the therapeutic effects of exercise-induced EVs. Another limitation of this study is the lack of a sedentary control group. The inclusion of a sedentary group in the study would provide an opportunity to investigate the anti-tumorigenic effects of acute exercise-induced circulating EVs on cancer cells as a function of the fitness levels of the participating individuals. Therefore, we strongly recommend that future studies include a sedentary control group. In addition, the study was conducted only in young males, and the effects of sex differences could not be examined. Conversely, given the average age of cancer patients, further investigation into the effects of exercise-induced EVs in the elderly is warranted.

In conclusion, our findings reveal for the first time that exercise-derived EVs from human participants reduce cell viability and induce apoptosis in HT-29 cells in an intensity-dependent manner. Further investigation of the EV cargo to identify biological factors, such as proteins, peptides, and miRNAs, will facilitate a deeper understanding of the therapeutic effects of exercise in prevention and potential treatment applications in cancer.

## Data Availability

Data is available from the primary author upon reasonable request.
